# Schedule-dependent therapeutic efficacy of L19mTNF-α and melphalan combined with gemcitabine

**DOI:** 10.1002/cam4.89

**Published:** 2013-05-29

**Authors:** Lorenzo Mortara, Paola Orecchia, Patrizia Castellani, Laura Borsi, Barbara Carnemolla, Enrica Balza

**Affiliations:** 1 Department of Biotechnology and Life Sciences, University of InsubriaVarese, Italy; 2 Department of Experimental Medicine, University of GenoaGenova, Italy; 3 Department of Translational OncologyLaboratory of Cell Biology, IRCSS AOU San Martino-Istituto Nazionale per la Ricerca sul CancroGenova, Italy; 4 Department of Translational OncologyLaboratory of Immunology, IRCSS AOU San Martino-Istituto Nazionale per la Ricerca sul CancroGenova, Italy

**Keywords:** Gemcitabine, immune response, L19mTNF-α, melphalan, mouse tumor models

## Abstract

L19-tumor necrosis factor alpha (L19mTNF-α; L), a fusion protein consisting of mouse TNFα and the human antibody fragment L19 directed to the extra domain-B (ED-B) of fibronectin, is able to selectively target tumor vasculature and to exert a long-lasting therapeutic activity in combination with melphalan (M) in syngeneic mouse tumor models. We have studied the antitumor activity of single L19mTNF-α treatment in combination with melphalan and gemcitabine (G) using different administration protocols in two histologically different murine tumor models: WEHI-164 fibrosarcoma and K7M2 osteosarcoma. All responding mice showed significant reduction in myeloid-derived suppressor cells (MDSCs) and an increase in CD4^+^ and CD8^+^ T cells in the tumor infiltrates, as well as significant reduction in regulatory T cells (Treg) at the level of draining lymph nodes. What is important is that all cured mice rejected tumor challenge up to 1 year after therapy. Targeted delivery of L19mTNF-α synergistically increases the antitumor activity of melphalan and gemcitabine, but optimal administration schedules are required. This study provides information for designing clinical studies using L19mTNF-α in combination with chemotherapeutic drugs.

Targeted delivery of L19mTNF-α synergistically increases the antitumor activity of melphalan and gemcitabine, but optimal administration schedule requires a pretreatment with L19mTNF-α otherwise an antagonistic effect could occur. This study provides information for designing clinical studies using L19mTNF-α in combination with chemotherapeutic drugs.

## Introduction

The extra domain-B (ED-B)–containing fibronectin isoform (B-FN) is a marker of angiogenesis 1–2 that can be employed to selectively target the tumor vasculature using a human recombinant antibody specific for B-FN, L19 (scFv), both in experimental animal models and in cancer patients 3–4. Tumor necrosis factor alpha (TNF-α) is a pleiotropic cytokine that exerts its antitumor effects mainly through a combination of preferential toxicity for tumor-associated endothelial cells and through an increase in the antitumor immune response. The toxicity for endothelial cells of the tumor vasculature results in extensive tumor necrosis 5–6. Furthermore, TNF-α increases vascular permeability 7 and reduces the tumor's interstitial fluid pressure 8 facilitating the penetration of antitumor agents at the tumor site. Although TNF-α is a potent antitumor cytokine, the associated toxicity prevents systemic administration at therapeutically effective doses. To circumvent this problem, we generated a L19mTNF-α fusion protein 9, with the murine TNF-α (mTNF-α) fused to the L19(scFv) antibody fragment.

A single systemic treatment with L19mTNF-α and melphalan (L-M) has been shown to be highly effective in two histologically unrelated mouse tumor models, WEHI-164 fibrosarcoma and C51 colon carcinoma, implanted in syngeneic BALB/c mice 10. Importantly, the combined therapy reduces regulatory T cells (Treg) in the tumor-draining lymph nodes and induces a long-lasting T-cell–mediated immune response against tumors involving both CD4^+^ and CD8^+^ T cells. This correlates with the capacity to reject new tumor challenges and metastases, even of different histological origin 10–11. Gemcitabine (2′,2′-difluorodeoxycytidine) is a nucleoside analog of cytidine, which acts as a single agent and in combination with cisplatin or other drugs against a wide range of solid tumors 12–16. Gemcitabine is considered appealing for combined therapies due to its mild toxicity profile at the active dose 17–18, and in recent years, several studies have shown that, when combined with other chemotherapeutic or immunotherapeutic compounds, it is more effective than when administrated alone 19,20. Preclinical studies have shown that gemcitabine treatment alone induces T lymphocyte expansion and tumor infiltration, increased tumor antigen cross-presentation, and significant reduction of Gr-1^+^CD11b^+^ myeloid-derived suppressor cells (MDSCs) in the spleens of tumor-bearing mice 20.

The accumulation of MDSCs 22 together with CD4^+^CD25^+^ Treg cells 23–24 in both animal models and in cancer patients is a key feature of tumor progression contributing to immune suppression with strong inhibition and dysfunction of antitumor CD4^+^ T-helper cells (Th) and CD8^+^ cytolytic T lymphocytes (CTL). Current immunotherapy approaches to treat cancer are aimed at triggering both Th and CTL tumor–specific immune responses 25 and at eliminating inherent suppressive mechanisms that can impact these cells 26.

In this report, by utilizing two syngeneic mouse tumor models, WEHI-164 fibrosarcoma and K7M2 osteosarcoma, we have compared the antitumor activity of L19mTNF-α/melphalan (L-M) and gemcitabine (G) as single therapies or combined treatments according to the different administration schedules. Moreover, we have evaluated the intratumoral infiltrates and the modulation of peripheral MDSCs and Treg cells in the draining lymph nodes induced by treatments**.**

## MATERIALS AND METHODS

### Animal tumor models

WEHI-164 mouse fibrosarcoma (3 × 10^6^) (ECACC, Sigma-Aldrich, Milan, Italy) and K7M2 mouse osteosarcoma (0.3 × 10^6^) (American Type Culture Collection, Rockville, MD), all BALB/c origin, were subcutaneously (s.c.) implanted in the left flank of 8- to 10-week-old immunocompetent syngeneic BALB/c mice, purchased from Harlan UK (Oxon, U.K.). Tumors were measured with a caliper and the tumor volume was determined using the following formula: (*d*)^2^ × *D* × 0.52, where *d* and *D* are the short and long dimensions (cm) of the tumor, respectively. The mice were sacrificed when the tumors reached a volume of about 1.5 cm^3^. The housing, treatment, and sacrifice of animals followed national legislative provisions (Italian Law no. 116 of 27 January 1992).

### Experimental protocol

When the tumors reached a volume of ~0.15 cm^3^, groups of 10 tumor-bearing mice received the therapeutic treatments as specified in Table 1A. Gemcitabine (G; Gemzar, Ely Lilly Italia S.P.A., Italy) was intraperitoneally (i.p.) administered at a dose of 120 mg/kg in 400 μL phosphate-buffered saline (PBS) (20 mmol/L NaH_2_PO_4_, 150 mmol/L NaCl, pH 7.4); L19mTNF-α [9] (L) 0.7 pmol/g was intravenously (i.v.) injected in 100 μL of PBS; melphalan (M; Alkeran, GlaxoSmithKline, Research Triangle Park, NC) was i.p. given at a dose of 4.5 mg/g in 400 μL of PBS. The animals' weight was recorded daily and weight loss never exceeded 5% within 72 h of the treatment. The tumor growth curves were recorded and the results of the treatment were expressed as a percentage of tumor-free survival versus time.

**Table:** Administration schedules of gemcitabine (G) and L19mTNF-α/melphalan (L-M) as single or combined treatments

Schedules	WEHI-164 (day)	K7M2 (day)
(A) Therapeutic treatments		
Tumor cell (s.c.)	0	0
G-G	+3/+10	+12/+19
L-M	+6^1^/+7	+15^1^/+16
G-L-M-G	+3/+6/+7/+10	+12/+15/+16/+19
L-M-G-G	+6/+7/+10/+17	+15/+16/+19/+26
(B) *in vivo* depletion protocol		
Anti-CD4^+^ (i.p.) Anti-CD8^+^ (i.p.)	0/+4/+8	+9/+14/+19

The therapeutic schedules (A) and *in vivo* depletion protocols (B) reported were applied on both tumor models at the times indicated.

1 Day at which tumor volume is ~0.15 cm^3^.

### *In vivo* T-cell subset depletion

*In vivo* depletions of T-cell subsets were performed as previously described 27 by three i.p. injections of anti-CD4 (GK1.5; ATTC, Rockville, MD) or anti-CD8 (2.43; ATTC) monoclonal antibodies (Table 1B). Control animals received irrelevant rat mAb, as described 27. Depletion efficiency for each cellular subset was monitored on splenocytes of two euthanized mice deriving from each group by using immunofluorescence and flow cytofluorimetric analysis (FACS) analysis (Becton Dickinson, Milan, Italy). Cytofluorimetric analysis was done by direct staining for CD4 fluorescein isothiocyanate (FITC-conjugated YTS 191.1.2 mAb; Immunotools, GmbH, Germany) or CD8 (PE-conjugated YTS 169.4 mAb; Immunotools) and was always >95%.

### Immunohistochemical analyses

Cryostat sections (6 μm thick) were air dried and fixed in cold acetone for 10 min. Immunostaining was performed as previously described 1. The following primary antibodies were used: anti-CD4 (clone GK1.5, ATCC), anti-CD8 (clone 2.43, ATCC); antigranulocyte Ly-6G (Gr-1; clone RB6–8C5), anti-CD11b (clone M1/70), and antimacrophage (clone MOMA1) were from Immunokontact (Oxon, U.K.); anti-CD45R (anti-B220 Ly5) was purchased from Southern Biotech (Birmingham, AL); anti-NK (antiasialo-GM1) was from Wako Chemicals (Dusseldorf, Germany). Quantitative studies of stained sections were performed independently by three researchers in a blinded fashion. Cell counting was carried out in 8–12 randomly chosen fields using a Leica Wetzlar light microscope (Germany) at 400× magnification, 0.180 mm^2^/field. The results are defined as cell number per high-magnification microscopic field (cell no./HMMF, mean ± SE).

### Adoptive immunity transfer experiments (Winn assay) and cell-mediated cytotoxicity

Six months post therapy, WEHI-164- and K7M2-cured mice were given a s.c. booster dose in the contralateral flank with cells derived from the same tumors (3 × 10^6^, WEHI-164; 0.3 × 10^6^, K7M2) and, within 12 days, the total splenocytes were obtained, following the procedure described by Mortara et al. 27, and used in a Winn assay at an effector:target (E:T) ratio of 1:1 for WEHI-164 tumor cells and an E:T ratio of 10:1 for K7M2 tumor cells. The results are specified as a percentage of tumor-free survival versus time. For cell-mediated cytotoxicity assay, we used splenocytes from tumor-cured mice 12 days after a tumor booster with the same tumor cells, 6  months post cure, as previously reported 28.

### Staining for MDSCs and Treg

The presence and the proportion of Gr-1^+^CD11b^+^ MDSCs and CD4^+^CD25^+^ Treg cells in the spleen and lymph nodes of naive, WEHI-164, and K7M2 tumor-bearing mice (treated and untreated) were assessed by FACS analysis using CELLQUEST software (Becton Dickinson). Cell suspensions were first incubated with anti-CD16 mAb to block Fc receptor, washed and stained with anti-CD4 FITC-conjugated and anti-CD25 PE-conjugated mAb (eBioscience, San Diego, CA). The expression of FoxP3, an additional marker of Tregs, was assessed in permeabilized cells using a FITC-labeled anti-FoxP3 rat mAb (clone FJK-16s; eBioscience). A FITC-labeled rat IgG2a mAb (eBioscience) was used as a negative control. In order to carry out analysis of MDSCs, splenic cell suspensions were first incubated with anti-CD16 mAb to block Fc receptor, stained with anti-Gr-1 (Ly-6G) FITC-conjugated and anti-CD11b-PE-conjugated mAb (eBioscience). A FITC- and PE-labeled rat IgG2b mAb (eBioscience) was used as a negative control.

### Statistical analysis

All results are presented as mean ± SD or SE for each group. Statistically significant differences between the groups were evaluated by nonparametric Kruskall–Wallis test using InStat Graph Pad for MAC. All *P*-values ≤0.001 and ≤0.01 were considered very significant and significant, respectively.

## Results

### Gemcitabine improves the antitumor efficacy of L19mTNF-α/melphalan

BALB/c mice–bearing WEHI-164 fibrosarcoma and K7M2 osteosarcoma were treated with G-G schedule or with L-M therapy, as indicated in Table 1A, when the s.c. tumors had the volume of about 0.15 cm^3^ (at day 6 for WEHI-164 and at day 15 for K7M2).

**Figure 1:**
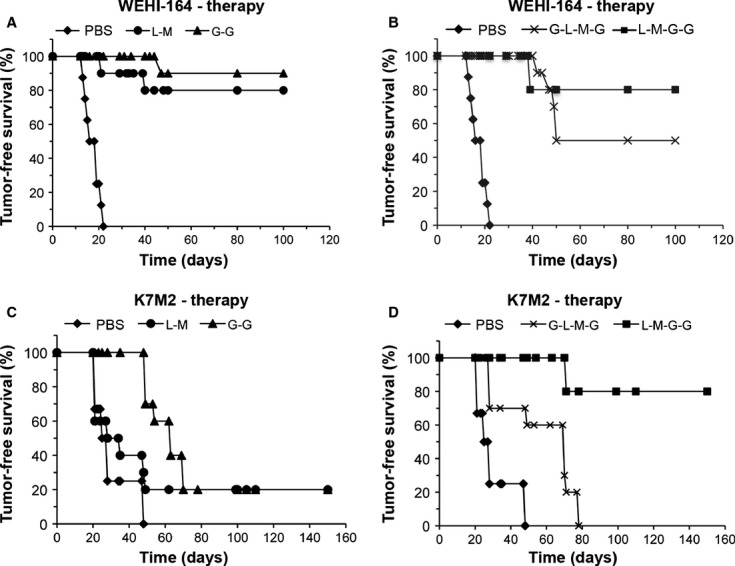
Therapeutic efficacy of the different schedules of drugs administration. Tumor-free survival curves (%) versus time (days) of WEHI-164 (A and B) and K7M2 (C and D) tumor-bearing mice subjected to G-G (black triangles), L-M (black circles), G-L-M-G (black asterisks), or L-M-G-G (black squares) administration schedules. Data are illustrative of at least 10 mice per treatment group.

As reported in Figure 1 (A and C) either G-G and L-M therapies determined complete and long-lasting tumor eradication in 80% or more of the highly cured WEHI-164 and in 20% of the poorly cured K7M2 tumor-bearing mice.

To improve upon the efficacy of gemcitabine or L19mTNF-α/melphalan therapies, we administered the three compounds in two different protocols (Table 1A). Noteworthy, when the protocol L-M-G-G was followed (Table 1A), the K7M2 tumor eradication jumped from 20% (obtained with L-M or G-G schedule) to 80% (Fig. 1D), revealing the highly synergistic effect of the three drugs. On the contrary, using a similar protocol, no enhanced therapeutic effect was observed in WEHI-164 tumor-bearing mice (Fig. 1B), where the results obtained with L-M or G-G schedule were already very high (more than 80% of tumor-free survival). Surprisingly, the protocol G-L-M-G (Table 1A), resulted in a drastic loss of therapeutic efficacy in both WEHI-164 and K7M2 tumor-bearing mice with respect to G-G or L-M treatments. In fact, a reduction in tumor-free survival from 80% to 50% for WEHI-164 and from 20% to 0% for K7M2 was observed with the G-L-M-G schedule (Fig. 1B and D).

### *In vivo* depletion of CD4^+^ or CD8^+^ T cells abolishes the antitumor efficacy of gemcitabine therapy

**Figure 2:**
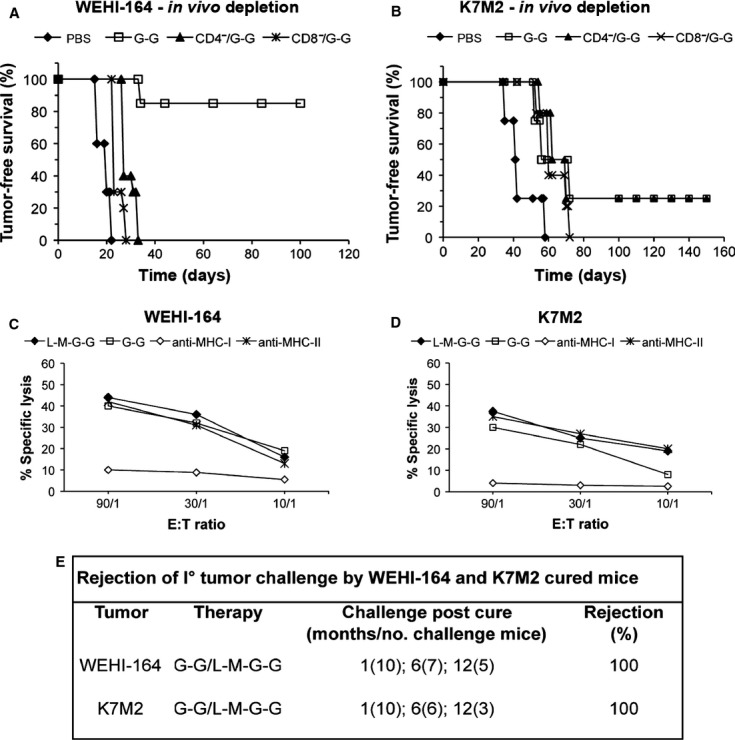
Immune population involved in WEHI-164 and K7M2 tumor eradication in *in vivo*-depleted mice. Tumor-free survival curves (%) versus time (days) of the WEHI-164 and K7M2 tumor-bearing mice subjected to G-G treatment, *in vivo* depleted with antibodies direct with CD4^+^ or CD8^+^ T cells as described in the Materials and Methods section. The tumor-free survival of groups of five WEHI-164 (A) and K7M2 (B) tumor-bearing mice subjected to G-G treatment (open squares), co-CD4-depleted (black triangles), co-CD8-depleted (black asterisks), or untreated tumor-bearing mice (black diamonds) is indicated. Specific cytolytic activity of the immune splenocytes after WEHI-164 and K7M2 tumor cure and persistence of antitumor memory. Specific lysis (%) of WEHI-164 (C) and K7M2 (D) cells by different E:T ratio of splenocytes from WEHI-164- and K7M2-cured mice at 6 months after G-G (open squares) or L-M-G-G (black diamonds) treatment and after s.c. tumor challenge. The specific lysis is totally inhibited by anti-MHC class I antibodies (open diamonds) and unaffected by anti-MHC class II antibodies (black asterisks). Results are representative of three independent ^51^Chromium-release experiments with similar results. (E) The ability of WEHI-164- and K7M2-cured mice subjected to G-G and L-M-G-G treatments to reject the first tumor challenge at different times post cure. The number of challenged mice is indicated in round brackets.

We previously demonstrated the key role played by the CD8^+^ and CD4^+^ T-cell compartments in tumor immune eradication of WEHI-164 tumor-bearing mice subjected to L-M therapy 11. So as to investigate the relative contribution of the two distinct lymphocyte subpopulations in the early processes leading to tumor eradication with the G-G schedule treatment, groups of 10 BALB/c mice were deprived of CD4^+^ or CD8^+^ T cells by specific mAb injection according to the protocol (Table 1B). Deprivation of CD8^+^ T cells completely abrogated the therapeutic effects of the G-G schedule treatment in both murine tumor models tested, thus demonstrating that CD8^+^ T cells are essential for tumor immune eradication (Fig. 2A and B). However, the removal of CD4^+^ T cells in WEHI-164 tumor-bearing mice totally abrogated the therapeutic effects of G-G schedule treatment, thus suggesting the importance of the CD4^+^ Th-cell compartment in the immune rejection of this tumor histotype, as was previously demonstrated in the case of L-M therapy 11 (Fig. 2A).

### Cured mice reject tumor challenges and demonstrate long-lasting immune memory

In order to study the antitumor cytolytic T-cell function, we evaluated the ability of total spleen cells from several WEHI-164 and K7M2 tumor-cured mice to kill homologous tumor cell lines (Fig. C and D). Six months post cure, splenocytes from WEHI-164- and K7M2-cured mice were assayed, against WEHI-164 and K7M2 tumor cell lines for in vitro cytotoxicity by using a ^51^Cr release standard cytotoxic assay (see Materials and Methods). A highly specific lysis at various E/T ratios on WEHI-164 and K7M2 target cells with G-G and the combined L-M-G-G treatments was observed. This lysis was MHC class I restricted as shown by strong inhibition of lysis in presence of anti-MHC class I mAb but not of anti-MHC class II mAb (Fig. 2C and D). Similar results after L-M therapy were obtained with splenocytes from K7M2-cured mice (data not shown) and WEHI-164-cured mice as previously demonstrated 11. The specific lysis was associated with fast and complete tumor rejection *in vivo* and was still present up to 1 year post treatment, thus demonstrating a very long-lasting antitumor immune memory (Fig. 2E). No tumor-specific CTL activity was found in the spleens from mice with growing WEHI-164 and K7M2 tumors, as well as from naive mice (data not shown). Winn assay experiments, performed with whole spleen cells 1 year post therapy clearly demonstrated that the induced effector cells were potent and long lasting because they were still able to protect 80–100% naive mice from homologous tumor cell challenges (data not shown).

### Modulation of the intratumoral infiltrating MDSCs, CD4^+^, and CD8^+^ T cells by single and combined treatments

**Figure 3:**
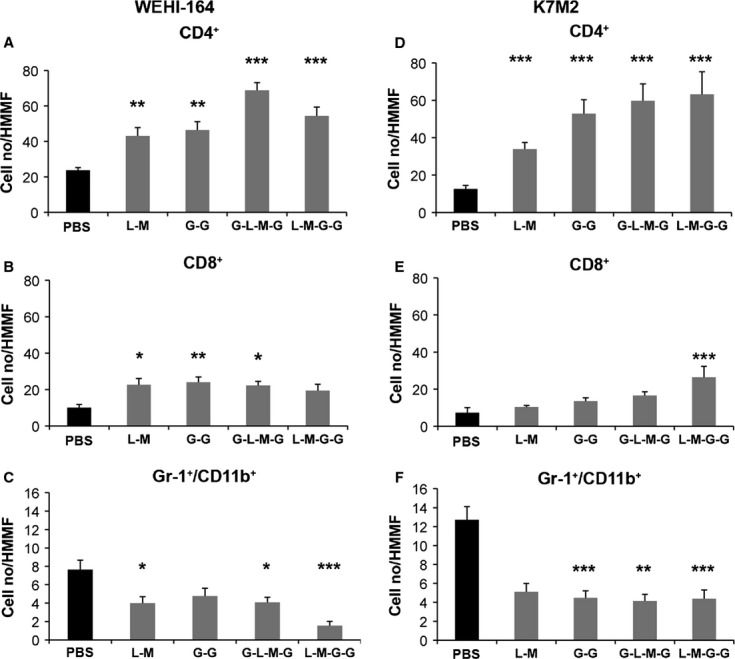
Immunohistochemical assessment of tumor infiltrates. Immunohistochemical assessment of CD4^+^ T cells, CD8^+^ T cells, and Gr-1^+^CD11b^+^ MDSCs in untreated or treated WEHI-164 (A–C) and K7M2 (D–F) tumor-bearing mice 3 days after the conclusion of all therapeutic protocols. Untreated group of mice received PBS only. Results are expressed as cell number (mean ± SD) per high-magnification microscopic field (HMMF). Data are representative of at least three mice per each treatment group. ***P ≤ 0.001; **P ≤ 0.01; *P ≤ 0.05.

To gain additional insight into the immune-mediated mechanisms associated with different administration protocols, we have investigated the phenotype of tumor-infiltrating leukocytes on cryostat sections of WEHI-164 and K7M2 tumor-bearing mice. Tumors were removed 3 days subsequent to the final administration of drugs for each schedule (Table 1A). Among the tumor-infiltrating cells analyzed, CD4^+^ T cells were statistically increased in WEHI-164 and K7M2 tumor-bearing mice which had been subjected to all protocol treatments, in comparison with untreated tumor–bearing mice (Fig. 3A and D). Significant increases in CD8^+^ T cells were found after the L-M, G-G, and G-L-M-G schedules in WEHI-164 tumors and after the L-M-G-G schedule in K7M2 tumors (Fig. 3B and E). Natural killer cells did not show any substantial increase in tumoral infiltrates as a result of therapy, as well as B cells and macrophages (data not shown).

**Figure 4:**
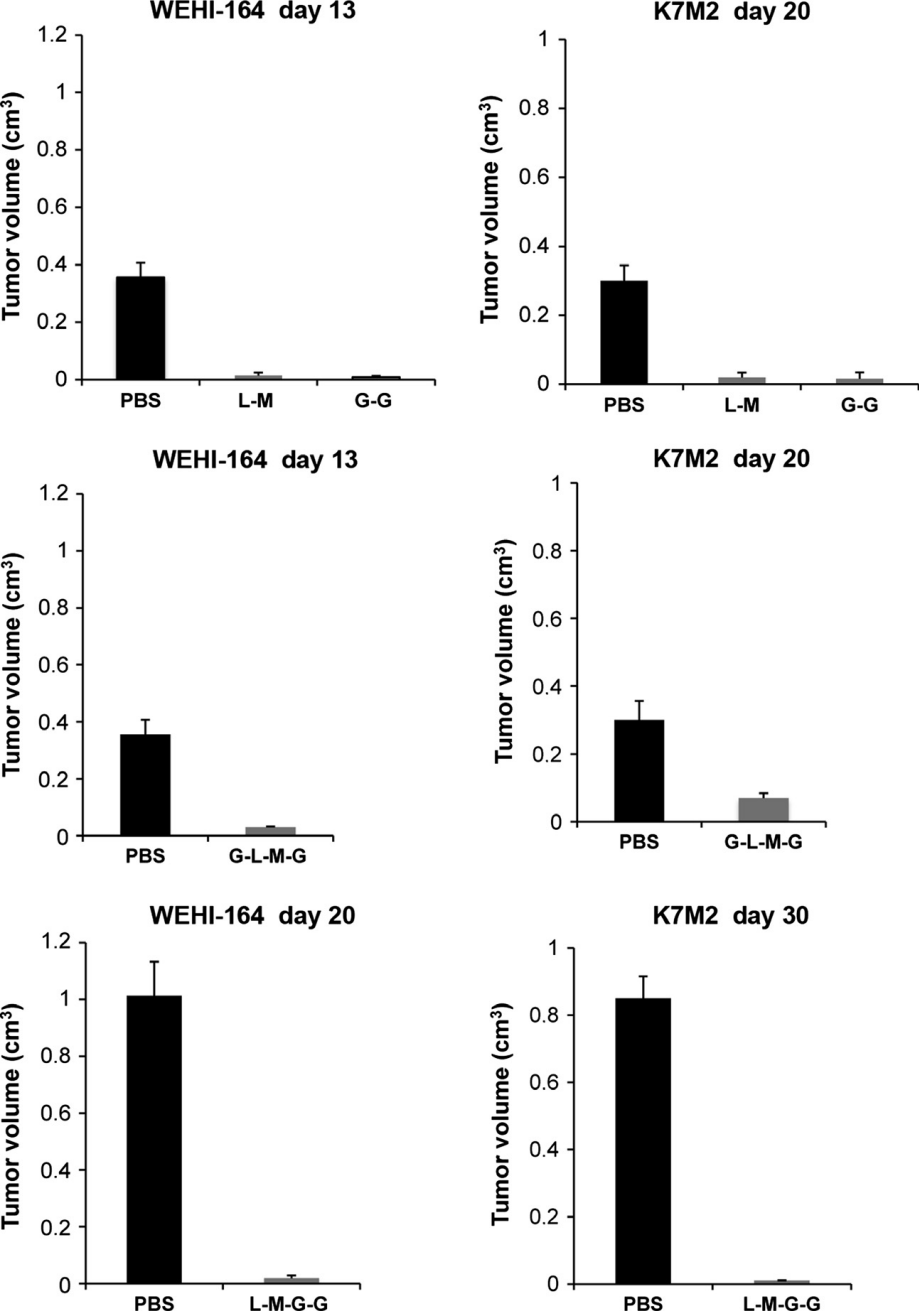
Tumor volume assessment after the different therapeutic treatments. Three days after the term of the all therapeutic protocols, the tumor volumes of WEHI-164 and K7M2 untreated and treated tumor-bearing mice were compared. Untreated mice received PBS only. Tumor volumes (cm^3^) are expressed as mean ± SD. Data are illustrative of least 10 mice per each treatment group.

In contrast, the infiltration of the MDSCs at the tumor site, particularly massive in untreated tumor–bearing mice, was statistically decreased in all treatment protocols, and in both tumor models with the exception of the G-G schedule in WEHI-164 fibrosarcoma and of the L-M schedule in K7M2 osteosarcoma (Fig. 3C and F). Notably, the decrease in the MDSCs and the increase in the CD4^+^ and CD8^+^ T cells correlated with drastic reduction in tumor volume observed after all therapeutic treatments (Fig. 4). The modulation of the CD4^+^, CD8^+^ T cells, and MDSCs infiltrating both tumors is shown in Figure S1A and B.

### Modulation of peripheral MDSCs and Treg cells in the draining lymph nodes by single and combined treatments

In order to assess the involvement of Treg cells and MDSCs in the process of tumor growth and in the antitumor immune response induced by the different drug administration protocols, both the quantitative variations in the draining lymph nodes and spleens of tumor-bearing mice were analyzed 3 days after the final treatment. All therapeutic protocols (Table 2) induced in the draining lymph nodes of both tumors showed a significant reduction in Treg cells, with respect to untreated mice. Unexpectedly, no significant reduction in the splenic MDSCs was observed in WEHI-164 and K7M2 tumor-bearing mice after all therapeutic protocols were tested (data not shown).

**Table:** Flow-cytometric assessment of regulatory T cells

Treatment	WEHI-164				K7M2						
	CD4^+^CD25^+^ (% ± SE)	FoxP3^+^CD25^+^ (% ± SE)	CD4^+^CD25^+^ cell no. (×10^6^) ± SE	CD4^+^CD25^+^ (% ± SE)	FoxP3^+^CD25^+^ (% ± SE)	CD4^+^CD25^+^ cell no. (×10^6^) ± SE
Naive	4.8 ± 0.7	4.38 ± 0.06	0.21 ± 0.04	4.8 ± 0.7	4.38 ± 0.06	0.21 ± 0.04
Untreated	6.0 ± 0.9	5.30 ± 0.2	0.37 ± 2.6	7.0 ± 0.2	5.90 ± 0.3	0.44 ± 0.3
G-G	4.9 ± 0.6	3.70 ± 0.5	0.06 ± 0.9***	4.6 ± 0.3	2.70 ± 0.2	0.29 ± 0.2***
L-M	4.1 ± 0.1	2.30 ± 0.9	0.09 ± 0.2***	3.7 ± 0.3	3.05 ± 0.5	0.28 ± 0.2***
G-L-M-G	4.3 ± 0.3	3.00 ± 0.2	0.06 ± 0.9***	5.1 ± 0.3	4.50 ± 0.1	0.32 ± 0.2***
L-M-G-G	2.4 ± 0.3	2.54 ± 0.8	0.08 ± 0.6***	4.1 ± 0.3	3.20 ± 0.5	0.21 ± 0.1***

CD4^+^CD25^+^ Treg cells assessment in tumor-draining lymph nodes after therapeutic treatments.

The modulation of CD4^+^CD25^+^ and FoxP3^+^CD25^+^ double-positive Treg cells from tumor-draining lymph nodes was evaluated in naive-treated or untreated WEHI-164 and K7M2 tumor-bearing mice 3 days after the term of all the therapeutic treatments. The results are indicated in percentage (%) form and absolute number (cell no.) (mean ± SE). Data are representative for at least three mice belonging to the treatment group.

*** ^*^^*^^*^P ≤ 0.001; ^*^^*^P ≤ 0.01; ^*^P ≤ 0.05.

## Discussion

Here, we found that treatment with gemcitabine or L19mTNF-α/melphalan induced a similar therapeutic effect in WEHI-164 (90% and 80% eradication, respectively) as well as in K7M2 (20% eradication) tumor-bearing mice (Fig. 1A and C). The combined treatment with all three compounds varied greatly with different administration protocols, indicating that the schedule sequence is a very important factor influencing the antitumor activity of the drug association. In fact, opposite results were obtained following G-L-M-G or L-M-G-G schedules in both tumors (Fig. 1B and D).

We then studied with *in vivo* cell-depletion experiments the role of CD8^+^ and CD4^+^ T cells in the tumor rejection phase and found the importance of CD8^+^ and of both CD4^+^ and CD8^+^ T lymphocyte subsets in the eradication of K7M2 (Fig. 2B) and WEHI-164 tumors (Fig. 2A), respectively. The cured mice developed a long-lasting antitumor immune memory that is able to reject a homologous tumor challenge up to 1 year after therapy (Fig. 2E) and to protect (Winn assay) 80%–100% naive mice from tumors (data not shown). These findings corroborate the idea that the curative protocols are able to generate a stable and protective antitumor immune cell reservoir that counteracts tumor take and growth after rechallenge.

In addition to their cytotoxic effects, gemcitabine and L19mTNF-α/melphalan therapies are known to mediate several relevant antitumor immunological effects. The main actions of gemcitabine are to augment antigen-specific cellular antitumor immunity 20 and to induce specific reduction in MDSCs 29 that is hypothesized to play a role in gemcitabine-mediated tumor regression 29–31. L19mTNF-α/melphalan therapy promotes the maturation of dendritic cells *in vivo* and their migration to draining lymph nodes 32, as well as the downmodulation of regulatory T cells 10–11 which are involved in the control of T-cell immune response and in the immune tolerance to tumors 23–36.

To evaluate the role of the peripheral MDSCs and Tregs in the draining lymph nodes in the antitumor immune response, we have detected their quantitative variations in WEHI-164 and K7M2 tumor-bearing mice, subjected to the different administration protocols. Our data highlighted the significant reduction in regulatory T cells in both tumor models, as compared with either untreated or naive mice, not specifically correlated with a particular administration schedule (Table 2). Furthermore, we did not observe a significant modulation in the absolute number of peripheral MDSCs (data not shown) with respect to the untreated tumor-bearing mice. These data indicate that all treatments were successful in reducing Treg cells, but rather less effective in decreasing MDSCs. Immunohistochemical results showed, in all treated tumors, a significant increase in the intratumoral CD4^+^ and CD8^+^ T cells that are the major lymphocyte populations involved in the antitumor priming and tumor rejection *in vivo* in addition to a significant decrease in the intratumoral MDSCs (Figs. 3 and S1). This result does not seem to correlate with the sharp increase in the tumor cure (jump from the 20% to the 80%) observed in K7M2 tumor-bearing mice subjected to L-M-G-G protocol with respect to G-L-M-G treatment.

At this regard, it is important to consider the time point we analyzed (3 days after the end of all treatments), as the results could vary at different observation times.

Interestingly, TNF-α particularly in the format of fusion proteins (like L19TNF-α) is able to target selectively angiogenic tumor vessels, to enhance the tumor penetration and to improve the antitumor therapeutic efficacy of chemotherapeutics-like melphalan 9–37 or gemcitabine 21. Therefore, we speculate that the strong antitumor effect showed by L-M-G-G schedule in K7M2 osteosarcoma is principally due to the efficacy of the vascular-targeted L19mTNF-α that induces massive tumor necrosis 11 with release of proinflammatory cytokines and chemokines 38, and tumor antigens that enhance the tumor immunogenicity and stimulate the activation of CD8^+^ T cells necessary for tumor eradication.

Moreover, the downmodulation of CD4^+^CD25^+^ Treg cells, induced by L-M therapy 11, may favor the therapeutic effect of gemcitabine and contribute to the overall cellular and molecular events leading to tumor eradication and stable antitumor immune response observed in these models.

In conclusion, our results, while confirming that gemcitabine per se stimulates and induces a curative acquisition of a long-lasting antitumor immunological memory 39, demonstrate that L19mTNF-α synergistically increases the antitumor activity of melphalan and gemcitabine in combined protocols that must be carefully designed to avoid dangerous antagonistic effects.

## Supporting Information

**Figure S1.** Immunohistochemical assessment of tumor infiltrates. Immunohistochemical assessment of CD4^+^ T cells, CD8^+^ T cells, and Gr-1^+^ CD11b^+^ MDSCs, respectively, in WEHI-164 (A) and K7M2 (B) tumor-bearing mice untreated (a–c), treated G-L-M-G (d–f), and L-M-G-G (g–i) 3 days after the conclusion of all therapeutic protocols. Untreated group of mice received PBS only. Magnification 400×.
