# Supplementary figures and images for: Schedule-dependent therapeutic efficacy of L19mTNF-α and melphalan combined with gemcitabine
Source: Cancer Med. 2013 May 29;2(4):478–87. doi: 10.1002/cam4.89 (PMC3799282; doi:10.1002/cam4.89)

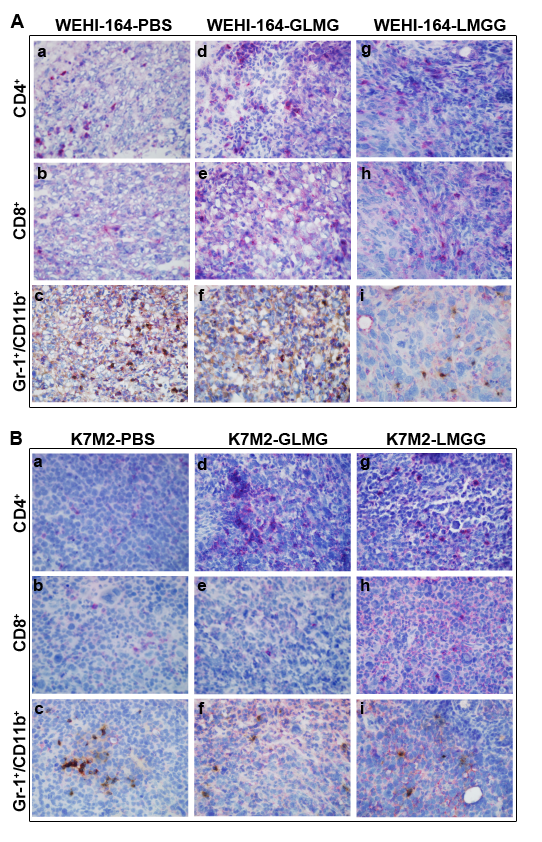

Supplement: Supplementary file 1 — Figure S1. Immunohistochemical assessment of tumor infiltrates. Immunohistochemical assessment of CD4+ T cells, CD8+ T cells, and Gr-1+ CD11b+ MDSCs, respectively, in WEHI-164 (A) and K7M2 (B) tumor-bearing mice untreated (a–c), treated G-L-M-G (d–f), and L-M-G-G (g–i) 3 days after the conclusion of all therapeutic protocols. Untreated group of mice received PBS only. Magnification 400×. [file cam40002-0478-SD1.tif]
